# Cutoffs on severity metrics for minimal manifestations or better status in patients with generalized myasthenia gravis

**DOI:** 10.3389/fimmu.2024.1502721

**Published:** 2024-12-23

**Authors:** Genya Watanabe, Yoshiki Takai, Yuriko Nagane, Tomoya Kubota, Manato Yasuda, Hiroyuki Akamine, Yosuke Onishi, Akiyuki Uzawa, Naoki Kawaguchi, Masayuki Masuda, Shingo Konno, Itaru Amino, Naoya Minami, Takashi Kimura, Makoto Samukawa, Takamichi Sugimoto, Yasushi Suzuki, Masanori P. Takahashi, Shigeaki Suzuki, Hiroyuki Murai, Masashi Aoki, Kimiaki Utsugisawa

**Affiliations:** ^1^ Department of Neurology, National Hospital Organization Sendai Medical Center, Sendai, Japan; ^2^ Department of Neurology, Tohoku University Graduate School of Medicine, Sendai, Japan; ^3^ Department of Neurology, Hanamaki General Hospital, Hanamaki, Japan; ^4^ Department of Clinical Laboratory and Biomedical Science, Osaka University, Suita, Japan; ^5^ Department of Neurology, Graduate School of Medicine, Chiba University, Chiba, Japan; ^6^ Neurological Center, Neurology Chiba Clinic, Chiba, Japan; ^7^ Department of Neurology, Tokyo Medical University, Tokyo, Japan; ^8^ Department of Neurology, Toho University Oh-hashi Medical Center, Tokyo, Japan; ^9^ Department of Neurology, National Hospital Organization Hokkaido Medical Center, Sapporo, Japan; ^10^ Department of Neurology, Hyogo Medical University, Nishinomiya, Japan; ^11^ Department of Neurology, Faculty of Medicine, Kindai University, Osakasayama, Japan; ^12^ Department of Clinical Neuroscience and Therapeutics, Hiroshima University, Hiroshima, Japan; ^13^ Department of Neurology, Keio University School of Medicine, Tokyo, Japan; ^14^ Department of Neurology, International University of Health and Welfare, Narita, Japan

**Keywords:** myasthenia gravis, receiver operating characteristic curve, minimal manifestations, cutoff value, treatment goal, myasthenia gravis foundation of America postintervention status

## Abstract

International consensus guidance and Japanese clinical guidelines for myasthenia gravis (MG) recommend achieving minimal manifestations or better status (MM-or-better) as the severity component of the treatment goal. However, the subjective nature of determining MM can result in ambiguity regarding this category in clinical practice and clinical trials. This study analyzed severity metrics in a large number of MG patients to propose criteria for MM-or-better. We utilized data obtained from 3800 MG patients who participated in nationwide cross-sectional surveys in Japan. Among these, 2784 patients with generalized MG were divided into two groups based on MG Foundation of America postintervention status: MM-or-better status (n = 1432); and improved-or-worse (I-or-worse) status (n = 1352). We compared severity metrics (MG-activities of daily living scale [MG-ADL], quantitative MG score [QMG], and MG composite scale [MGC]) between groups and calculated cutoff values to separate the two groups. Using these cutoffs, patients subjectively assigned as MM-or-better were classified into strict MM-or-better (below a cutoff) or optimistic MM-or-better (above a cutoff) groups, and clinical characteristics were then compared. Cutoff values for strict MM-or-better were MG-ADL ≤2, QMG ≤7, and MGC ≤4 (sensitivity 82.0%, 88.7%, and 87.4%; specificity 85.0%, 70.0%, and 77.9%; and accuracy 91.2%, 88.7%, and 90.7%, respectively). Mean values of the revised 15-item MG quality of life scale were significantly lower in the strict MM-or-better group than in the optimistic MM-or-better group. Quantitative criteria for MM-or-better appear likely to be useful in the context of rigorous clinical trials and also as reference information in clinical settings.

## 1 Introduction

In the treatment of generalized myasthenia gravis (gMG), high-dose chronic oral steroid therapy has reduced the mortality and frequency of severe disease since the 1970s (1–3), but many gMG patients have still been living with poor health-related quality of life (QOL) due to an inability to sufficiently reduce oral steroid doses and unstable MG symptoms caused by inadequate therapeutic intervention (3–6). New treatment strategies (7–9) and targeted drugs (10–14) have recently been developed to further improve QOL for gMG patients. Given that the rate of full remission from MG remains low even today (1, 5, 7, 8), setting achievable and appropriate treatment goals to optimize QOL is important.

Treatment goals for MG often comprise components for both improvement status and burden due to treatment (7, 8, 15–19). International consensus guidance (18, 19) and Japanese clinical guidelines (7, 8, 16, 17) for MG together indicate a component of MG symptoms for the initial treatment goal as: achieving a state of minimal manifestations or better (MM-or-better) in the MG Foundation of America postintervention status (MGFA-PIS). In addition to achieving MM-or-better, the Japanese clinical guidelines for MG recommend reducing the oral prednisolone (PSL) dose to ≤5 mg/day (termed “MM-5 mg”) as soon as possible (8), whereas international consensus guidance for MG encourage goals of MM-or-better with no more than grade 1 Common Terminology Criteria for Adverse Events medication side effects (18, 19).

MM is defined as the status of no symptoms with functional limitations from MG, but some weakness on examination of some muscles (20). Ultimately, in patient-centered clinical settings it should be up to the patient to decide whether they have symptoms that interfere with a normal lifestyle, where MM do not necessarily mean a ‘MG activities of daily living scale (MG-ADL) (21) score of 0 point’. MM is a realistic and achievable treatment goal that can lead to good QOL for patients, given that complete stable remission (CSR) and pharmacologic remission (PR) are frequently difficult to achieve. However, the subjective nature of MM may lead to ambiguity in goal setting in clinical practice. To avoid such ambiguity, employing some objective reference values for MM-or-better may prove helpful. Regarding endpoints in rigorous clinical trials, criteria for MGFA-PIS status have been suggested to require definition in each study protocol based on quantitative assessments (20). Demonstrating quantitative criteria for MM could therefore be useful for determining MM in the context of rigorous clinical trials and also as supplementary information in clinical settings.

In this study, scores from several MG severity scales such as the MG-ADL (21), quantitative myasthenia gravis score (QMG) (22), and myasthenia gravis composite scale (MGC) (23) from 2784 patients with gMG were analyzed to determine significant cutoff values for MM-or-better and to provide objective criteria for evaluating the clinical severity of MM-or-better. Furthermore, using these cutoffs, we classified patients assigned subjectively as MM-or-better into strict MM-or-better (below a cutoff) or optimistic MM-or-better (above a cutoff) groups, compared clinical characteristics between the groups and reported their implications in rigorous trials and clinical settings.

## 2 Materials and methods

### 2.1 Patients

Our cross-sectional multi-center surveys were conducted in 2010, 2012, 2015, and 2021 at 20 neurological centers in total [Japan MG registry (JAMG-R) study]. In each of these surveys, consecutive and established MG patients with various stages of illness over a short duration (4 months) were all enrolled to avoid potential bias. Our study was based on accurate reports by motivated neurologists (not on reports from patients), with many detailed data all quantified and entered on special case cards created in Claris FileMaker Pro^®^ (Claris International Inc., California, The United States), which were then directly expanded and integrated into a large Excel^®^ file (Microsoft Corporation, Washington, The United States) and used as the source of the database for the analyses. Input errors and missing values were corrected or re-surveyed through discussion between the secretariat (Y.N.) and the neurologist in charge. We confirm that we have read the Journal’s position on issues involved in ethical publication and affirm that this report is consistent with those guidelines. The ethics committees of each participating institution approved the study protocols. Written informed consent was obtained from all patients enrolled in the study.

Clinical information obtained routinely in our surveys included: sex, current age, age at onset, duration of disease, duration from onset to start of immunotherapy administration, presence of bulbar symptoms, history of MG crisis, presence of AChR-Ab or MuSK-Ab, history of thymectomy and thymic histology, current dose and maximum dose of PSL, use of non-steroid oral immunosuppressants such as calcineurin inhibitors (CNIs), plasmapheresis, and/or intravenous immunoglobulin (IVIg), MGFA classification at worst condition of the disease, current clinical status according to MGFA-PIS, severity scores at current and worst condition, body mass index (BMI), and QOL of patients (15-item myasthenia gravis quality of life scale [MG-QOL15]). Clinical severity was determined according to the MG-ADL, QMG, and MGC. Due to the coronavirus disease 2019 (COVID-19) pandemic, use of a spirometer was avoided, and QMG was not evaluated in the 2021 survey. Regarding QMG, only data obtained in 2010 (1st survey), 2012 (2nd survey) and 2015 (3rd survey) were available.

**Figure 1:**
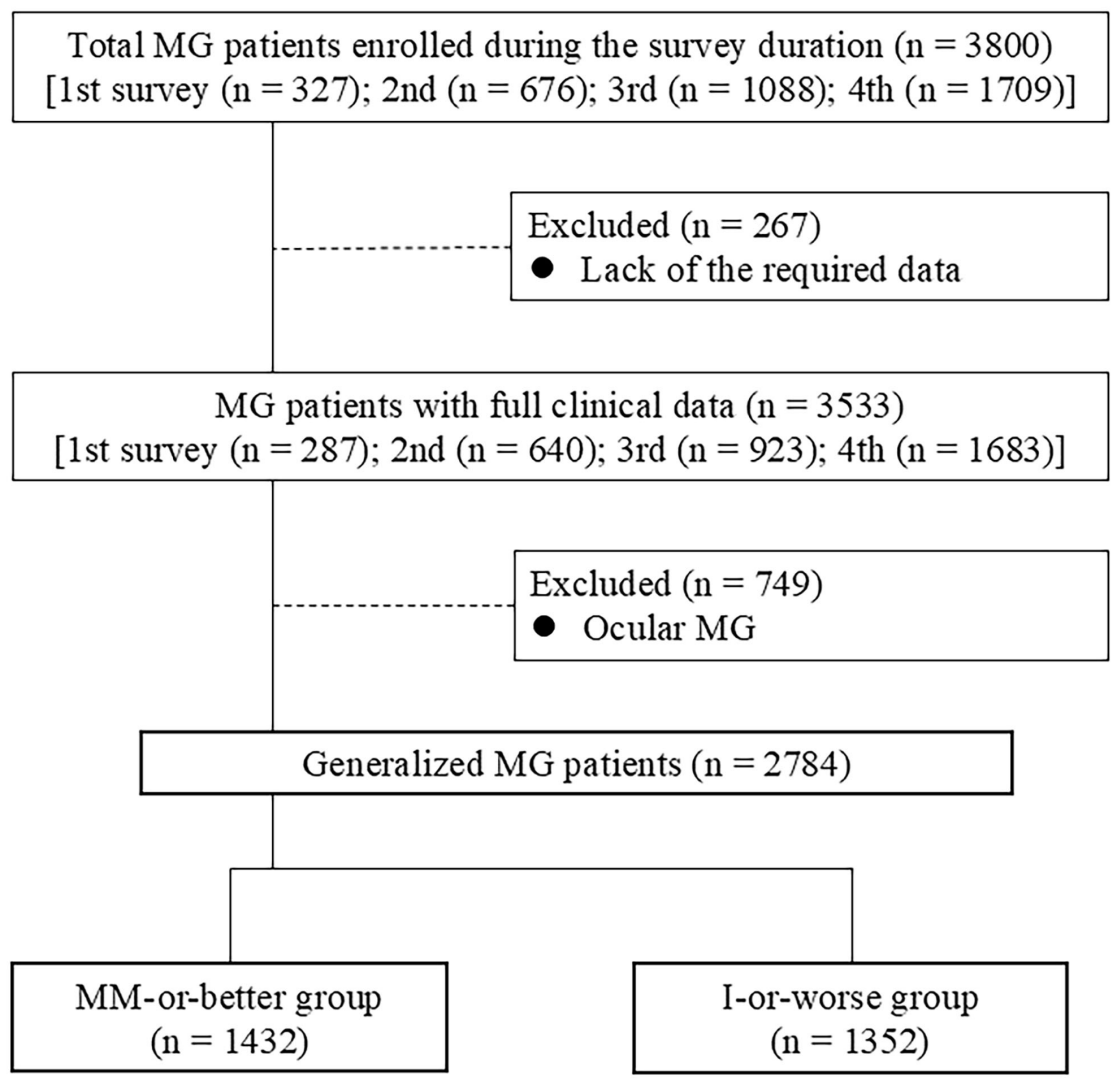
CONSORT diagram of study participants; *I*, improved status; *MM*, minimal manifestations; *MG*, myasthenia gravis.

Data from 3800 patients in total were collected from a series of four surveys in 2010, 2012, 2015, and 2021 (**Figure 1**). Of those, 267 were excluded due to missing data from the MGFA-PIS, MG-ADL, QMG, and/or MGC. In total, data from 3533 patients, representing a real number of 2486 patients (428 patients enrolled in two surveys, 143 patients in three surveys, and 111 patients in all four surveys), were analyzed (287 patients from the 2010 survey (3, 6), 640 patients from the 2012 survey (24), 923 patients from the 2015 survey (9), and 1683 from the 2021 survey). Of these 3533 patient data, 749 patients with ocular MG were excluded, given that levels of severity scores corresponding to MM-or-better naturally differ between patients with ocular MG and gMG. The remaining 2784 patients with gMG were divided into two groups: 1432 patients with MM-or-better status (achieving CSR, PR, or MM); and 1352 patients with I-or-worse status [not achieving MM or better status; status of improved (I), unchanged (U), worse (W), or exacerbation (E) in MGFA-PIS] (20).

Diagnosis of MG was performed according to the diagnostic criteria set forth in the 2014 Japanese clinical guideline for MG (8, 16): In brief, the diagnosis was based on clinical findings (fluctuating muscle symptoms with easy fatigability and recovery after rest) and the presence of antibodies against skeletal muscle acetylcholine receptor (AChR-Ab) or muscle-specific tyrosine kinase (MuSK-Ab), or, when neither AChR-Ab nor MuSK-Ab was detected, on clinical findings and clinical improvement after administration of anticholinesterase, decremental muscle responses to a 3-Hz train of repetitive nerve stimuli (25) and/or an eyelid ice pack test with thorough exclusion of other diseases. A therapeutic diagnosis by response to plasma exchange was considered to confirm the diagnosis. Single-fiber electromyography was not performed systematically. AChR-Ab were measured by radioimmunoassay, using ^125^I-α-bungarotoxin ≥ 0.3 nmol/L considered positive (5) and MuSK-Ab were measured via radioimmunoprecipitation assay, with a value ≥ 0.02 nmol/L considered positive (26). In Japanese clinical guidelines, low-density lipoprotein receptor-related protein (LRP) 4 antibody-positivity using current assay systems are not considered diagnostic findings due to reasons including a lack of disease specificity (17), and therefore are not measured systematically but are only measured in antibody-negative cases as a reference finding. 

The MG-QOL15 is a 15-item patient-administered questionnaire, with each item scored from 0 to 4 points, for a maximum total score of 60 points (27). The revised MG-QOL15 (MG-QOL15r) is a simplified version of the MG-QOL15, with very similar questions that have a 0–2 points distribution and a maximum total score of 30 points (28). As both the original and revised MG-QOL15 were taken as measures of QOL at different time points in our survey, a conversion value of total MG-QOL15 score/60 or total MG-QOL15r score/30 was used to create a corrected MG-QOL15 (cMG-QOL15) for analysis.

### 2.2 Strict and optimistic MM-or-better

Clinically assigned MM-or-better patients (n = 1432) were classified into two groups using the individual cutoff values for three severity metrics (MG-ADL, QMG, and MGC): the strict MM-or-better group and the optimistic MM-or-better group, with severities below or above the cutoff level. Clinical characteristics were compared between strict and optimistic MM-or-better groups on each scale. Due to the COVID-19 pandemic, use of a spirometer was avoided, and QMG score was not evaluated in the 2021 survey. Therefore, regarding the strict/optimistic MM-or-better group on QMG score, data for a total of 745 patients from the 2010, 2012, and 2015 surveys were analyzed.

### 2.3 Statistical analysis

Comparisons between two groups were performed using the Mann–Whitney U test for continuous variables. Categorical variables were analyzed using Fisher’s exact test to compare the two groups, and the chi-square test to compare the three or more groups. Multiple comparisons for analyses of outcome measures were corrected using Bonferroni correction, in which the level of statistical significance is divided by the trial number of univariate analyses between the two groups, resulting in p-values < .002 on **Table 1** as statistical significance, <.004 on **Table 2**, and <.003 on **Table 3**. Receiver operating characteristic (ROC) curves were used to determine the ideal cutoff values for the MG-ADL, QMG, and MGC. The cutoff point on the ROC curve statistically corresponded to the point at which sensitivity - (1 - specificity) is maximal. This point was the maximum Youden index, which was used as the optimality criterion in cutoff point selection. Sensitivity and specificity values according to the optimal cutoff attributed to the Youden index were identified. The area under the ROC curve (AUC), which measures the ability of a binary classifier to distinguish between groups, was determined to evaluate the discrimination ability of the cutoff points. AUC with 95% confidence interval (CI) was reported. JMP Pro 15 statistical software^®^ (SAS Institute Inc., North Carolina, The United States) was employed for the analysis, and a value of p <.05 was considered to indicate statistical significance. For CIs, the lower and upper limits were analyzed from bias-corrected confidence limits based on the bootstrap method at the level of p <.05.

**Table 1:** Comparison of demographic data between MM-or-better and I-or-worse groups (Mann–Whitney U-test).

	MM-or-better group (n = 1432)	I-or-worse group (n = 1352)	*p* value
Sex, male/female (female%) *	527/905 (63.2)	368/984 (72.8)	<.0001^†^
Age, years, mean (SD)	58.8 (16.7)	58.1 (16.0)	0.1314
Onset age, years, mean (SD)	47.1 (18.5)	45.9 (18.0)	0.0500
Disease duration, years, mean (SD)	12.9 (9.50)	13.4 (11.1)	0.7493
Duration to start of immunotherapy, years, mean (SD)	1.82 (4.14)	2.29 (4.93)	<.0001^†^
Bulbar symptoms, n (%) *	866 (60.5)	965 (71.4)	<.0001^†^
History of MG crisis, n (%) *	134 (9.36)	158 (11.7)	0.0477
EOMG/LOMG/TAMG, % **	37.5/33.2/29.3	46.9/27.1/26.0	<.0001^†^
AChR-Ab positivity, n (%) *	1256 (87.7)	1030 (76.2)	<.0001^†^
MuSK-Ab positivity, n (%) *	20 (1.40)	34 (2.51)	0.1259
Thymoma, n (%) *	420 (29.3)	352 (26.0)	0.0566
Thymectomy, n (%) *	841 (58.7)	727 (53.8)	0.0093
Current MG-ADL, mean (SD)	1.27 (1.41)	5.70 (3.37)	<.0001^†^
Current QMG, mean (SD)	4.19 (2.86)	10.8 (5.04)	<.0001^†^
Current MGC, mean (SD)	1.85 (2.31)	9.11 (6.18)	<.0001^†^
Current cMG-QOL15, mean (SD)	0.15 (0.17)	0.41 (0.23)	<.0001^†^
Worst MGFA class (II/III/IV/V), % **	62.4/22.3/5.87/9.36	47.7/32.7/7.89/11.7	<.0001^†^
Maximum dose of PSL, mg, mean (SD)	25.4 (19.7)	24.6 (18.9)	0.4857
Current dose of PSL, mg, mean (SD)	3.41 (3.77)	5.81 (5.73)	<.0001^†^
CNI use, n (%) *	829 (57.9)	959 (70.9)	<.0001^†^
IVIg use, n (%) *	239 (16.7)	479 (35.4)	<.0001^†^
Plasmapheresis use, n (%) *	467 (32.6)	476 (35.2)	0.1495
Body mass index, mean (SD)	23.1 (3.99)	23.2 (4.57)	0.8041

*MG*, myasthenia gravis; *MM-or-better*, minimal manifestations-or-better status; *I-or-worse*, improved-or-worse status; *AChR-Ab*, anti-acetylcholine receptor antibody; *CNI*, calcineurin inhibitor; *EOMG*, early-onset myasthenia gravis; *IVIg*, intravenous immunoglobulin at 0.4 g/kg/day for 5 days; *LOMG*, late-onset myasthenia gravis; *MG-ADL*, myasthenia gravis activities of daily living scale; *MGC*, myasthenia gravis composite scale; *MGFA*, Myasthenia Gravis Foundation of America; *MuSK-Ab*, anti-muscle-specific kinase antibody; *PSL*, prednisolone; *QMG*, quantitative myasthenia gravis score; *SD*, standard deviation; *cMG-QOL15*, corrected 15-item myasthenia gravis quality of life scale; *TAMG*, thymoma-associated myasthenia gravis. *Fisher’s exact test, **Chi-square test, ^†^p <.002 for Bonferroni correction.

**Table 2:** Differences between strict MM-or-better and optimistic MM-or-better groups on MG severity scales.

	Strict MM-or-better	Optimistic MM-or-better	*p* value
Number of patients on MG-ADL (%)	1174 (82.0)	258 (18.0)	
Number of patients on QMG (%)	661 (88.7)	84 (11.3)	
Number of patients on MGC (%)	1251 (87.4)	181 (12.6)	
Number of patients on cMG-QOL15 (%)	1068 (74.6)	364 (25.4)	
Sex, male/female (female%), on MGC	487/764 (61.1)	41/140 (77.3)	<.0001^†^
Age, years, mean (SD), on MG-ADL	57.8 (16.8)	63.6 (14.9)	<.0001^†^
Current MG-ADL, mean (SD, range), on MG-ADL	0.74 (0.80, 0-2)	3.67 (1.01, 3-8)	<.0001^†^
Current QMG, mean (SD, range), on QMG	3.50 (2.10, 0-7)	9.62 (2.11, 8-16)	<.0001^†^
Current MGC, mean (SD, range), on MGC	1.16 (1.37, 0-4)	6.56 (1.96, 5-14)	<.0001^†^
Current cMG-QOL15, mean (SD, range), on MG-ADL	0.12 (0.15, 0-0.8)	0.27 (0.23, 0-1.2)	<.0001^†^
CNI use, n (%), on MG-ADL *	699 (59.5)	129 (50.0)	0.0054
Plasmapheresis use, n (%), on MG-ADL *	412 (35.1)	55 (21.3)	<.0001^†^
Worst QMG, mean (SD), on MGC	13.5 (6.47)	15.7 (7.03)	0.0004^†^

*MG*, myasthenia gravis; *MM-or-better*, minimal manifestations-or-better status; *MG-ADL*, myasthenia gravis activities of daily living scale; *CNI*, calcineurin inhibitors; *MGC*, myasthenia gravis composite scale; *QMG*, quantitative myasthenia gravis score; *SD*, standard deviation; *cMG-QOL15*, corrected 15-item myasthenia gravis quality of life scale; Range represents between minimum and maximum values, *Fisher’s exact test, ^†^p <.004 for Bonferroni correction.

**Table 3:** Comparison between gMG patients achieving minimal symptom expression group and achieving strict MM-or-better on the MG-ADL scale (Mann–Whitney U-test).

	Achieving MSE (n = 914)	Achieving strict MM-or-better (n = 1174)	*p* value
Sex, n (%), male/female (female%) *	355/559 (61.2)	443/731 (62.3)	0.6177
Age, years, mean (SD)	57.6 (17.1)	57.8 (16.8)	0.8740
Disease duration, years, mean (SD)	12.2 (8.99)	12.6 (9.25)	0.2859
EOM disturbance, n (%) *	669 (73.2)	877 (74.7)	0.4506
History of MG crisis, n (%) *	87 (9.51)	112 (9.54)	1.0000
AChR-Ab positivity, n (%) *	788 (86.2)	1026 (87.4)	0.4338
Thymoma, n (%) *	286 (31.3)	349 (29.7)	0.4434
Current QMG, mean (SD)	3.67 (2.57)	3.90 (2.63)	0.1601
Current MGC, mean (SD)	0.91 (1.57)	1.33 (1.88)	<.0001^†^
Current cMG-QOL15, mean (SD)	0.10 (0.13)	0.12 (0.15)	0.0006^†^
Worst MGFA class, % **			0.9915
II	62.8	62.4	
III	21.9	22.0	
IV	5.75	6.10	
V	9.51	9.54	
Maximum dose of PSL, mg, mean (SD)	25.7 (19.8)	25.6 (19.8)	0.9683
Current dose of PSL, mg, mean (SD)	3.37 (3.71)	3.41 (3.78)	0.8922
CNI use, n (%) *	551 (60.3)	699 (59.5)	0.7528
IVIg use, n (%) *	152 (16.6)	197 (16.8)	0.9529
Plasmapheresis use, n (%) *	339 (37.1)	412 (35.1)	0.3581

*gMG*, generalized myasthenia gravis; *MG-ADL*, myasthenia gravis activities of daily living scale; *MM-or-better*, minimal manifestations-or-better status; *AChR-Ab*, anti-acetylcholine receptor antibody; *CNI*, calcineurin inhibitor; *EOM*, external ocular movement; *IVIg*, intravenous immunoglobulin at 0.4 g/kg/day for 5 days; *MG*, myasthenia gravis; *MGC*, myasthenia gravis composite scale; *MGFA*, Myasthenia Gravis Foundation of America; *MSE*, minimal symptom expression; *PSL*, prednisolone; *QMG*, quantitative myasthenia gravis score; *SD*, standard deviation; *cMG-QOL15*, corrected 15-item myasthenia gravis quality of life scale; *Fisher’s exact test, ** chi-square test, ^†^p <.003 for Bonferroni correction.

## 3 Results

### 3.1 MM-or-better cutoff values based on each severity rating scale

Comparison of demographic data between MM-or-better and I-or-worse groups showed that the MM-or-better group had a significantly lower frequency of women, a shorter time to initiation of immunotherapy, and a higher rate of positivity for AChR-Ab (**Table 1**). Naturally, current MG-ADL, QMG, MGC, and cMG-QOL15 were significantly lower in the MM-or-better group than in the I-or-worse group. Regarding treatment in the MM-or-better group, current PSL dose and frequency of CNI use were lower, maximum dose of PSL and frequency of plasmapheresis did not differ significantly, and frequency of IVIg use was lower when compared to the I-or-worse group. 

**Figure 2:**
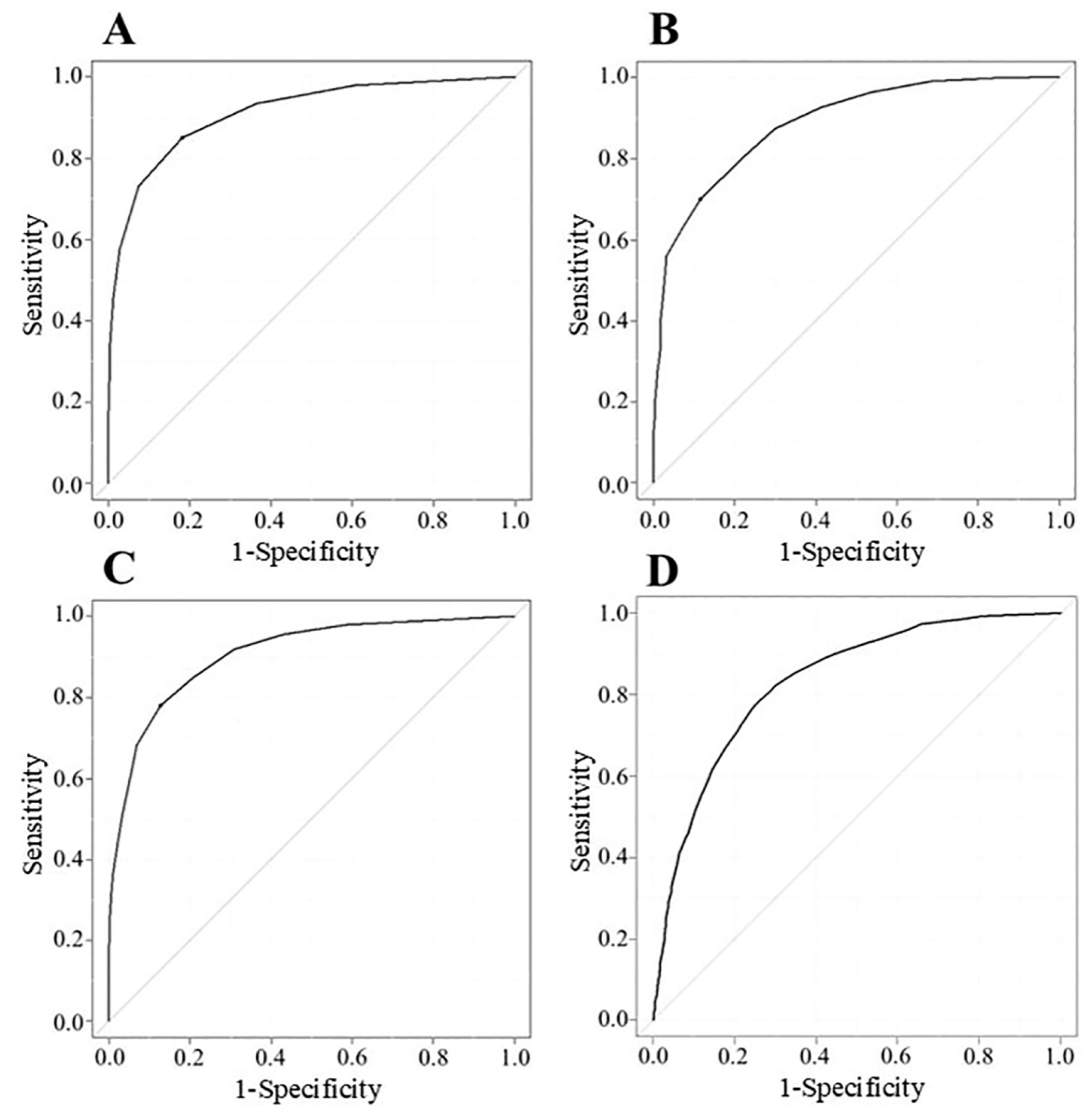
ROC curve described with data of MG-ADL (n = 2784) **(A)**, QMG (n = 1473) **(B)**, MGC (n = 2784) **(C)**, and cMG-QOL15 (n = 2784) **(D)**. Vertical axis shows sensitivity (true positive); horizontal axis shows 1-specificity (false positive). Cutoff points correspond to the point on the ROC curve where sensitivity - (1 - specificity) is maximal; *cMG-QOL15*, corrected 15-item myasthenia gravis quality of life scale; *MG-ADL*, myasthenia gravis activities of daily living scale; *MGC*, myasthenia gravis composite scale; *QMG*, quantitative myasthenia gravis score; *ROC*, receiver operating characteristic.

To analyze cutoff points between MM-or-better and I-or-worse groups on MG scores, ROC curves were drawn for MG-ADL, QMG, MGC, and cMG-QOL15, respectively. (**Figures 2A–D**). The AUC for MG-ADL, QMG, MGC and cMG-QOL15 were 0.912, 0.888, 0.906 and 0.832 respectively (**Table 4**). Based on the ROC curve, cutoff values were analyzed as follows: for MG-ADL, a cutoff of 2 points offered 82.0% sensitivity and 85.0% specificity; for QMG, a cutoff of 7 points offered 88.7% sensitivity and 70.0% specificity; for MGC, a cutoff of 4 points offered 87.4% sensitivity and 77.9% specificity; for cMG-QOL15, a cutoff of 0.2 points offered 74.6% sensitivity and 77.8% specificity (**Table 4**).

**Table 4:** Cutoff values, and sensitivity and specificity of MG severity metrics between MM-or-better and I-or-worse groups.

	Generalized MG (n = 2784)			
	MM-or-better group (n = 1432) vs. I-or-worse group (n = 1352)			
	Sens., %	Spec., %	Cutoff	AUC (95%CI)
MG-ADL	82.0	85.0	2	0.912 (0.900-0.923)
QMG*	88.7	70.0	7	0.888 (0.870-0.906)
MGC	87.4	77.9	4	0.906 (0.895-0.918)
cMG-QOL15	74.6	77.8	0.2	0.832 (0.816-0.847)

*MG*, myasthenia gravis; *MM-or-better*, minimal manifestations-or-better status; *I-or-worse*, improved-or-worse status; *cMG-QOL15*, corrected 15-item myasthenia gravis quality of life scale; *MG-ADL*, myasthenia gravis activities of daily living scale; *MGC*, myasthenia gravis composite scale; *QMG*, quantitative myasthenia gravis score; *Sens.*, sensitivity; *Spec.*, specificity; *Cutoff*, cutoff value; *AUC*, area under the curve; *CI*, confidence interval. *QMG has been compared between 745 patients with generalized MG in the MM-or-better group and 728 patients in the I-or-worse group.

To examine the impact of overlapping data, we created another dataset (n=1936) by deleting the older data from duplicate patients (first data for double duplicates, first and second data for triple duplicates, and first, second and third data for quadruple duplicates), and analyzed it. The analyzed data corresponding to **Tables 1**, **4** are shown in **Supplementary Tables 2, 3**, which demonstrated almost the identical results between 2784 (**Tables 1**, **4**) and 1936 (**Supplementary Tables 2, 3**) patients.

All three scale cutoffs were achieved in 622 (42.5%) of the 1462 patients with gMG from the 1st survey to the 3rd survey (patients within cutoffs of both MG-ADL and QMG, n = 695 [47.5%]; QMG and MGC, n = 725 [49.6%]; MG-ADL and MGC, n = 673 [46.0%]).

### 3.2 Difference between strict MM-or-better and optimistic MM-or-better

**Figure 3:**
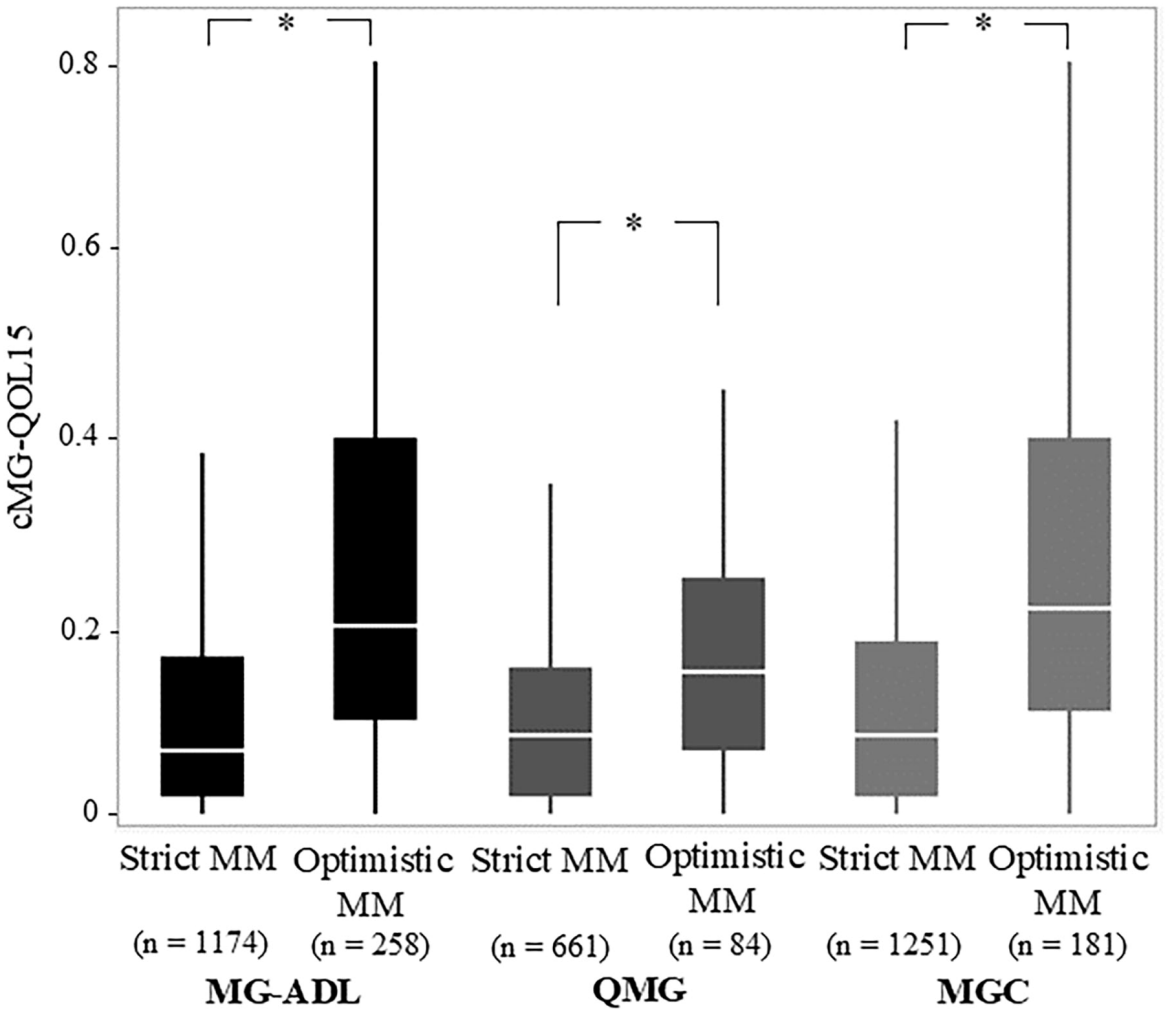
Comparison of Strict and Optimistic MM-or-better with cutoffs on MG-ADL, QMG, and MGC regarding corrected 15-items myasthenia gravis quality of life scale (cMG-QOL15). *MG-ADL*, myasthenia gravis activities of daily living scale; *MGC*, myasthenia gravis composite scale; *MM*, minimal manifestations; *QMG*, quantitative myasthenia gravis score; Mann–Whitney U-test; *p <.0001.

Common characteristics of the optimistic MM-or-better group across all cutoffs naturally included significantly higher current MG-ADL, current QMG, and current MGC compared with the strict MM-or-better group (**Table 2**). Specifically, current cMG-QOL15, which reflects patient QOL, was significantly higher (worse QOL) in the optimistic MM-or-better group than in the strict MM-or-better group when differentiated with severity scores (**Figure 3**, p <.0001; with MG-ADL cutoff, 0.12 ± 0.15 vs. 0.27 ± 0.23; with QMG cutoff, 0.11 ± 0.13 vs. 0.18 ± 0.13; with MGC cutoff, 0.13 ± 0.16 vs. 0.28 ± 0.22). In addition, optimistic MM-or-better patients based on MG-ADL cutoff were less frequently treated with CNIs and plasmapheresis and included a higher proportion of elderly patients, compared to strict MM-or-better patients (**Table 2**; **Supplementary Table 4**). Optimistic MM-or-better patients based on the QMG cutoff showed a significantly higher frequency of female patients, a higher proportion of MGFA III and V at worst condition, and higher QMG score at worst condition (**Supplementary Table 5**). Optimistic MM-or-better patients based on the MGC cutoff showed a higher proportion of females, a higher proportion of elderly patients, and higher QMG at worst condition (**Table 2**; **Supplementary Table 6**).

### 3.3 Comparisons between patients with strict MM-or-better on the MG-ADL Scale and minimal symptom expression

Among patients with gMG clinically assigned as strict MM-or-better (n = 1174), 914 patients achieved minimal symptom expression (MSE), defined as scoring 0–1 points on the MG-ADL scale (29). Differences between the group achieving MSE (MG-ADL 0–1) and the group achieving strict MM-or-better (MG-ADL 0–2) on the MG-ADL scale are shown in **Table 3**. Mean MGC were significantly lower in the MSE group than in the strict MM-or-better group, whereas mean QMG did not differ significantly between groups and actual differences in cMG-QOL15 were small (0.10 ± 0.13 vs. 0.12 ± 0.15, respectively) regardless of significance, due to the large sample size.

## 4 Discussion

The present study analyzed data from 2784 gMG patients in MM-or-better (n = 1432) and I-or-worse (n = 1352) groups, and demonstrated cutoff values for MM-or-better on individual severity scales, as follows: ≤ 2 points on MG-ADL; ≤ 7 points on QMG; and ≤ 4 points on MGC. On the other hand, in actuality, some patients clinically assigned as MM-or-better showed severity scores above these cutoffs (258/1432, 18.0% for the MG-ADL cutoff; 84/745, 11.3% for the QMG cutoff; and 181/1432, 12.6% for the MGC cutoff representing optimistic MM-or-better patients). Such MM-or-better patients appeared to be more frequent for the MG-ADL cutoff than for the QMG or MGC cutoffs. Considering the merits of patient-driven severity evaluation, the MG-ADL has often been employed as a primary endpoint in recent clinical trials for new therapies against gMG as well as in clinical settings (10–14). Since MG-ADL has no physician-driven evaluation item (27), use of cutoffs from QMG and MGC scores may be helpful as objective reference values in determining MM.

We proposed the idea of “strict” MM-or-better (within a severity cutoff) or “optimistic” MM-or-better (above the severity cutoff). In rigorous clinical trials, employing strict MM-or-better as a goal or endpoint may be more suitable to avoid ambiguity in determining MM. On the other hand, in clinical settings, optimistic MM-or-better should not be ignored as a means of attaching importance to the perspective of the patient. However, when assigning a patient to optimistic MM-or-better, re-evaluating the judgement of MM using the present cutoff values while taking into account the following issues may be better. In the present study, optimistic MM-or-better patients showed characteristics such as older age, higher frequency of females, and more severe disease in worst condition than strict MM-or-better patients (**Table 2**). Levels of MG symptoms considered as incurring ‘no limitation to a normal lifestyle’ by the individual patient may display a wide upper limit among elderly and/or female patients with experience of severe disease. Such patients may misunderstand that marked improvement in MG symptoms compared with the previous severe condition is a status of non-interference with daily life (i.e., MM-or-better), even if they still experience residual symptoms affecting good daily life. In their daily lives, the amount of activity might have already been limited in order to adapt to the symptoms. Furthermore, in this study, optimistic MM-or-better patients on the MG-ADL scale were less frequently treated with plasmapheresis and/or CNIs (**Table 2**), suggesting the possibility that they might have not received sufficient treatment.

Some goal statuses of MG treatment reflected in MG-specific severity metrics have been reported, such as patient-acceptable symptom state (PASS), as well as MSE (30). The PASS index represents a state in which the patient feels they are adequately well (31, 32). Cutoff values for PASS were demonstrated to be MG-ADL ≤ 2, QMG ≤ 7, MGC ≤ 3, and MG-QOL15 ≤ 8 (31). Thus, the cutoff values for strict MM-or-better shown for Japanese gMG patients (MG-ADL scale ≤ 2, QMG score ≤ 7, and MGC ≤ 4) were largely in line with those for PASS. Furthermore, it is reported that all (100%) of PASS positive patients had been simultaneously evaluated as MM-or-better (remission or MM) (31). The levels of MG symptoms at which the individual patient feels a status of non-interference with daily life may be universal on these severity metrics, regardless of ethnicity or geographic region, such as in Canada and Japan (31, 32). Strict MM-or-better and PASS can thus be considered a much practical treatment goal or endpoint as the severity component.

Recently, achieving MSE (defined as 0–1 points on the MG-ADL scale or a MG-QOL15r of 0–3) is often employed as a secondary outcome parameter in clinical trials (29). Regarding the MG-ADL scale, two items for ocular symptoms, in which ptosis and diplopia are each rated as 1 point even when not occurring every day, are difficult to discriminate between patients with and without MG symptoms interfering with daily life (21, 33, 34). These include infrequent modest ocular symptoms, which may not have an impact on QOL (33, 34). In addition, particularly among elderly patients, the effects of age- and/or comorbidity-related fatigue and frailty can affect MG-ADL scoring to some extent, even when the symptoms are unrelated to MG (35–38). The cutoff of ≤ 1 point on the MG-ADL scale for MSE may be too strict for use as a treatment goal at least in clinical settings, and in actuality, the achievement rate has not been high in clinical trials for new drugs (e.g. 14% for the zilucoplan group in RAISE study and 21.4% for the eculizumab group in REGAIN study) (10, 29). In the present study, MGC in the patient group achieving MSE (MG-ADL scale ≤ 1) were naturally lower than those in patients achieving strict MM-or-better (MG-ADL scale ≤ 2). However, QMG did not differ significantly between groups, and regarding cMG-QOL15, little numerical difference was evident.

Limitations of this study include the retrospective, cross-sectional nature, and the lack of data related to QMG score in 2021. However, we accumulated exact severity score data for a large number of cases from four cross-sectional surveys conducted from 2010 to 2021. In each of those four surveys, consecutive MG patients over a short duration were enrolled to avoid potential bias, and examined and reported by motivated neurologists. The present sample is probably one of the largest to date. Among the total of four surveys conducted, some patient overlap was seen. However, even if the present database included some data from the same patients, this had little impact on our analysis (**Supplementary Tables 2, 3**). The present findings do not necessarily mean that strict MM-or-better is a better category than PASS or MSE as a treatment goal, but we believe that the results are meaningful and useful for determining MM-or-better. In this study, data were collected from Japanese MG patients. However, the cutoff values for strict MM-or-better in the present study were largely in line with those for PASS as reported from Canada (31). Large differences in the levels of acceptable symptoms for patients on MG-specific severity metrics do not seem to exist among regions or ethnicity.

In conclusion, we have demonstrated that cutoff values for strict MM-or-better on MG-specific severity metrics were MG-ADL ≤ 2, QMG ≤ 7, and MGC ≤ 4. However, we do not believe that these cutoffs should be strictly used to determine MM in clinical settings. MG treatment and efficacy evaluation should be patient-centered, and the cutoffs may thus be useful as reference values. To avoid ambiguity in determining MM-or-better in rigorous settings such as clinical trials, employing the ideas of strict MM-or-better as needed may prove helpful. Multicenter studies in various regions are warranted to investigate objective MM indicators that can be applied globally.

## Data Availability

The raw data supporting the conclusions of this article will be made available by the authors, without undue reservation.
