# Supplementary material for: Cutoffs on severity metrics for minimal manifestations or better status in patients with generalized myasthenia gravis
Source: Front Immunol. 2024 Dec 23;15:1502721. doi: 10.3389/fimmu.2024.1502721 (PMC11701239; doi:10.3389/fimmu.2024.1502721)
Supplement: Supplementary file 1 [file Table1.docx]

**Supplementary Table 1**. Institutions participating in the Japan Myasthenia Gravis Registry study in 2021 survey

| Department of Neurology, National Hospital Organization Hokkaido Medical Center, Sapporo |
| --- |
| Department of Neurology, Hanamaki General Hospital, Hanamaki |
| Department of Neurology, National Hospital Organization Sendai Medical Center, Sendai |
| Neurological Center, Neurology Chiba Clinic, Chiba |
| Department of Neurology, Graduate School of Medicine, Chiba University, Chiba |
| Department of Neurology, International University of Health and Welfare, Narita |
| Department of Neurology, Keio University School of Medicine, Tokyo |
| Department of Neurology, Tokyo Medical University, Tokyo |
| Department of Neurology, Toho University Oh-hashi Medical Center, Tokyo |
| Department of Neurology, Osaka University Graduate School of Medicine, Suita |
| Department of Neurology, Kindai University Faculty of Medicine, Osakasayama |
| Department of Neurology, Hyogo Medical University, Nishinomiya |
| Department of Clinical Neuroscience and Therapeutics, Hiroshima University, Hiroshima |
