# Supplementary material for: Cutoffs on severity metrics for minimal manifestations or better status in patients with generalized myasthenia gravis
Source: Front Immunol. 2024 Dec 23;15:1502721. doi: 10.3389/fimmu.2024.1502721 (PMC11701239; doi:10.3389/fimmu.2024.1502721)
Supplement: Supplementary file 2 [file Table2.docx]

**Supplementary Table 2.** Comparison of demographic data between MM-or-better and I-or-worse groups in real number of patients without duplicate data (Mann–Whitney U-test)

|  | MM-or-better group  (n = 983) | I-or-worse group  (n = 953) | *p* value |
| --- | --- | --- | --- |
| Sex, male/female (female%) * | 367/616 (62.7) | 253/700 (73.5) | <.0001^†^ |
| Age, years, mean (SD) | 60.0 (16.5) | 58.6 (15.9) | 0.0321 |
| Onset age, years, mean (SD) | 47.6 (18.5) | 45.9 (18.2) | 0.0319 |
| Disease duration, years, mean (SD) | 12.7 (9.94) | 13.1 (11.5) | 0.4716 |
| Duration to start of immunotherapy, years, mean (SD) | 1.69 (3.82) | 2.36 (5.37) | 0.0006^†^ |
| Bulbar symptoms, n (%) * | 586 (59.6) | 669 (70.2) | <.0001^†^ |
| History of MG crisis, n (%) * | 92 (9.38) | 114 (12.0) | 0.0656 |
| EOMG/LOMG/TAMG, % ** | 36.1/32.3/31.6 | 46.2/27.4/26.4 | <.0001^†^ |
| AChR-Ab positivity, n (%) * | 870 (88.5) | 708 (74.3) | <.0001^†^ |
| MuSK-Ab positivity, (%) n * | 20 (2.03) | 33 (3.46) | 0.0693 |
| Thymoma, n (%) * | 311 (31.6) | 252 (26.4) | 0.0123 |
| Thymectomy, n (%) * | 570 (58.0) | 476 (49.9) | 0.0004 |
| Current MG-ADL, mean (SD) | 1.38 (1.45) | 6.07 (3.41) | <.0001^†^ |
| Current QMG, mean (SD) | 4.46 (2.95) | 11.7 (4.80) | <.0001^†^ |
| Current MGC, mean (SD) | 1.90 (2.34) | 9.29 (5.96) | <.0001^†^ |
| Current cMG-QOL15, mean (SD) | 0.16 (0.19) | 0.43 (0.24) | <.0001^†^ |
| Worst MGFA class (II/III/IV/V), % ** | 63.7/22.0/4.92/9.38 | 48.4/33.0/6.63/12.0 | <.0001^†^ |
| Maximum dose of PSL, mg, mean (SD) | 24.9 (19.5) | 23.9 (18.4) | 0.4989 |
| Current dose of PSL, mg, mean (SD) | 3.42 (3.75) | 6.13 (5.53) | <.0001^†^ |
| CNI use, n (%) * | 564 (57.4) | 680 (71.4) | <.0001^†^ |
| IVIg use, n (%) * | 190 (19.3) | 368 (38.6) | <.0001^†^ |
| Plasmapheresis use, n (%) * | 263 (26.8) | 305 (32.0) | 0.0125 |
| Body mass index, mean (SD) | 23.2 (4.16) | 23.2 (4.73) | 0.3340 |

*MG*, myasthenia gravis; *MM-or-better*, minimal manifestations-or-better status; *I-or-worse*, improved-or-worse status; *AChR-Ab*, anti-acetylcholine receptor antibody; *CNI*, calcineurin inhibitor; *EOMG*, early-onset myasthenia gravis; *IVIg*, intravenous immunoglobulin at 0.4 g/kg/day for 5 days; *LOMG*, late-onset myasthenia gravis; *MG-ADL*, myasthenia gravis activities of daily living scale; *MGC*, myasthenia gravis composite scale; *MGFA*, Myasthenia Gravis Foundation of America; *MuSK-Ab*, anti-muscle-specific kinase antibody; *PSL*, prednisolone; *QMG*, quantitative myasthenia gravis score; *SD*, standard deviation; *cMG-QOL15*, corrected 15-item myasthenia gravis quality of life scale; *TAMG*, thymoma-associated myasthenia gravis. *** Fisher’s exact test, **** Chi-square test, ^†^p < .002 for Bonferroni correction.
