# Supplementary material for: Cutoffs on severity metrics for minimal manifestations or better status in patients with generalized myasthenia gravis
Source: Front Immunol. 2024 Dec 23;15:1502721. doi: 10.3389/fimmu.2024.1502721 (PMC11701239; doi:10.3389/fimmu.2024.1502721)
Supplement: Supplementary file 3 [file Table3.docx]

**Supplementary Table 3**. Cutoff values, and sensitivity and specificity of MG severity metrics between MM-or-better and I-or-worse groups in real number of patients without duplicate data

|  | Generalized MG (n = 1936) | | | |
| --- | --- | --- | --- | --- |
|  | MM-or-better group (n = 983) vs. I-or-worse group (n = 953) | | | |
|  | Sens., % | Spec., % | Cutoff | AUC (95%CI) |
| MG-ADL | 79.6 | 88.8 | 2 | 0.921 (0.909-0.933) |
| QMG* | 88.1 | 78.7 | 7 | 0.905 (0.878-0.928) |
| MGC | 87.0 | 79.8 | 4 | 0.910 (0.897-0.924) |

*MG*, myasthenia gravis; *MM-or-better*, minimal manifestations-or-better status; *I-or-worse*, improved-or-worse status; *MG-ADL*, myasthenia gravis activities of daily living scale; *MGC*, myasthenia gravis composite scale; *QMG*, quantitative myasthenia gravis score; *Sens.*, sensitivity; *Spec.*, specificity; *Cutoff*, cutoff value; *AUC*, area under the curve; *CI,* confidence interval. *QMG has been compared between 258 patients with generalized MG in the MM-or-better group and 283 patients in the I-or-worse group.
