# Supplementary material for: Cutoffs on severity metrics for minimal manifestations or better status in patients with generalized myasthenia gravis
Source: Front Immunol. 2024 Dec 23;15:1502721. doi: 10.3389/fimmu.2024.1502721 (PMC11701239; doi:10.3389/fimmu.2024.1502721)
Supplement: Supplementary file 4 [file Table4.docx]

**Supplementary Table 4**. Comparison between strict MM-or-better and optimistic MM-or-better groups on MG-ADL scale

|  | Strict  MM-or-better  (n = 1174) | Optimistic  MM-or-better  (n = 258) | *p* value |
| --- | --- | --- | --- |
| Sex, male/female (female%) | 443/731 (62.3) | 85/173 (67.1) | 0.1546 |
| Age, years, mean (SD) | 57.8 (16.8) | 63.6 (14.9) | <.0001^†^ |
| Onset age, years, mean (SD) | 46.4 (18.6) | 50.2 (18.2) | 0.0042 |
| Disease duration, years, mean (SD) | 12.6 (9.25) | 14.1 (10.5) | 0.0612 |
| Time to beginning immunotherapy, years, mean (SD) | 1.72 (4.03) | 2.33 (4.63) | 0.8220 |
| Bulbar symptoms, n (%) * | 714 (60.8) | 151 (58.5) | 0.5270 |
| MG crisis onset, n (%) * | 112 (9.54) | 23 (8.91) | 0.8148 |
| EOMG/LOMG/TAMG, % ** | 37.9/32.4/29.7 | 35.8/37.0/27.2 | 0.3953 |
| AChR-Ab positivity, n (%) * | 1026 (87.4) | 229 (88.8) | 0.6022 |
| MuSK-Ab positivity, n (%) * | 15 (1.28) | 4 (1.55) | 0.7653 |
| Thymoma, n (%) * | 349 (29.7) | 70 (27.1) | 0.4499 |
| Thymectomy, n (%) * | 684 (58.3) | 156 (60.5) | 0.5304 |
| Current QMG, mean (SD) | 3.90 (2.63) | 6.38 (3.46) | <.0001^†^ |
| Current MGC, mean (SD) | 1.33 (1.88) | 4.21 (2.61) | <.0001^†^ |
| Current cMG-QOL15, mean (SD) | 0.12 (0.15) | 0.27 (0.23) | <.0001^†^ |
| Worst MGFA class, (II/III/IV/V), % ** | 62.4/22.0/6.10/9.54 | 63.3/22.9/4.80/8.91 | 0.8300 |
| Maximum dose of PSL, mg, mean (SD) | 25.6 (19.8) | 24.4 (19.4) | 0.3737 |
| Current dose of PSL, mg, mean (SD) | 3.41 (3.78) | 3.38 (3.70) | 0.9156 |
| CNI use, n (%) * | 699 (59.5) | 129 (50.0) | 0.0054 |
| IVIg use, n (%) * | 197 (16.8) | 42 (16.3) | 0.9266 |
| Plasmapheresis use, n (%) * | 412 (35.1) | 55 (21.3) | <.0001^†^ |
| Worst QMG, mean (SD) | 13.7 (6.44) | 14.0 (7.14) | 0.9665 |
| Worst MGC, mean (SD) | 15.7 (10.1) | 16.7 (11.5) | 0.7158 |

*MG*, myasthenia gravis; *MM-or-better*, minimal manifestations-or-better status; *MG-ADL*, myasthenia gravis activities of daily living scale; *AChR-Ab*, anti-acetylcholine receptor antibody; *CNI*, calcineurin inhibitors; *EOMG*, early-onset myasthenia gravis; *IVIg*, intravenous immunoglobulin at 0.4 g/kg/day for 5 days; *LOMG*, late-onset myasthenia gravis; *MGC*, myasthenia gravis composite scale; *MGFA*, Myasthenia Gravis Foundation of America; *MuSK-Ab*, anti-muscle-specific kinase antibody; *PSL*, prednisolone; *QMG*, quantitative myasthenia gravis score; *SD*, standard deviation; *cMG-QOL15*, corrected 15-item myasthenia gravis quality of life scale; *TAMG*, thymoma-associated myasthenia gravis. *** Fisher’s exact test, **** chi-square test, ^†^p < .002 for Bonferroni correction.
