# Supplementary material for: Cutoffs on severity metrics for minimal manifestations or better status in patients with generalized myasthenia gravis
Source: Front Immunol. 2024 Dec 23;15:1502721. doi: 10.3389/fimmu.2024.1502721 (PMC11701239; doi:10.3389/fimmu.2024.1502721)
Supplement: Supplementary file 5 [file Table5.docx]

**Supplementary Table 5**. Comparison between strict MM-or-better and optimistic MM-or-better groups on QMG score

|  | Strict  MM-or-better  (n = 661) | Optimistic  MM-or-better  (n = 84) | *p* value |
| --- | --- | --- | --- |
| Sex, male/female (female%) | 238/423 (64.0) | 12/72 (85.9) | <.0001^†^ |
| Age, years, mean (SD) | 56.8 (16.6) | 60.6 (16.6) | 0.0737 |
| Onset age, years, mean (SD) | 46.1 (18.5) | 48.4 (18.3) | 0.3245 |
| Disease duration, years, mean (SD) | 13.2 (9.13) | 14.8 (8.98) | 0.1754 |
| Time to beginning immunotherapy, years, mean (SD) | 2.15 (4.94) | 1.80 (2.87) | 0.5899 |
| Bulbar symptoms, n (%) * | 395 (59.7) | 56 (66.7) | 0.2379 |
| MG crisis onset, n (%) * | 59 (8.93) | 16 (19.0) | 0.0066 |
| EOMG/LOMG/TAMG, % ** | 38.3/34.1/27.7 | 42.3/33.8/23.9 | 0.7664 |
| AChR-Ab positivity, n (%) * | 567 (85.8) | 74 (88.1) | 0.7378 |
| MuSK-Ab positivity, n (%)* | 10 (1.51) | 2 (2.38) | 0.4543 |
| Thymoma, n (%) * | 183 (27.7) | 20 (23.8) | 0.5163 |
| Thymectomy, n (%) * | 398 (60.2) | 56 (66.7) | 0.2860 |
| Current MG-ADL, mean (SD) | 0.86 (1.09) | 2.06 (1.84) | <.0001^†^ |
| Current MGC, mean (SD) | 1.37 (1.85) | 4.59 (3.36) | <.0001^†^ |
| Current cMG-QOL15, mean (SD) | 0.11 (0.13) | 0.18 (0.13) | <.0001^†^ |
| Worst MGFA class, (II/III/IV/V), % ** | 62.7/21.5/6.90/8.93 | 54.0/25.6/1.43/19.0 | 0.0186 |
| Maximum dose of PSL, mg, mean (SD) | 26.2 (20.1) | 22.2 (18.4) | 0.1150 |
| Current dose of PSL, mg, mean (SD) | 3.60 (3.92) | 3.04 (3.63) | 0.2958 |
| CNI use, n (%) * | 386 (58.4) | 44 (52.4) | 0.2943 |
| IVIg use, n (%) * | 72 (10.9) | 8 (9.52) | 0.8520 |
| Plasmapheresis use, n (%) * | 270 (40.8) | 22 (26.2) | 0.0091 |
| Worst QMG, mean (SD) | 14.0 (6.65) | 17.7 (8.16) | 0.0023 |
| Worst MGC, mean (SD) | 15.6 (9.89) | 17.8 (13.7) | 0.9446 |

*MG*, myasthenia gravis; *MM-or-better*, minimal manifestations-or-better status; *QMG*, quantitative myasthenia gravis score; *AChR-Ab*, anti-acetylcholine receptor antibody; *CNI*, calcineurin inhibitor; *EOMG*, early-onset myasthenia gravis; *IVIg*, intravenous immunoglobulin at 0.4 g/kg/day for 5 days; *LOMG*, late-onset myasthenia gravis; *MG-ADL*, myasthenia gravis activities of daily living scale; *MGC*, myasthenia gravis composite scale; *MGFA*, Myasthenia Gravis Foundation of America; *MuSK-Ab*, anti-muscle-specific kinase antibody; *PSL*, prednisolone; *SD*, standard deviation; *cMG-QOL15*, corrected 15-item myasthenia gravis quality of life scale; *TAMG*, thymoma-associated myasthenia gravis. *** Fisher’s exact test, **** chi-square test, ^†^p < .002 for Bonferroni correction.
