# Supplementary material for: Cutoffs on severity metrics for minimal manifestations or better status in patients with generalized myasthenia gravis
Source: Front Immunol. 2024 Dec 23;15:1502721. doi: 10.3389/fimmu.2024.1502721 (PMC11701239; doi:10.3389/fimmu.2024.1502721)
Supplement: Supplementary file 6 [file Table6.docx]

**Supplementary Table 6**. Comparison between strict MM-or-better and optimistic MM-or-better groups on MGC scale

|  | Strict  MM-or-better  (n = 1251) | Optimistic  MM-or-better  (n = 181) | *p* value |
| --- | --- | --- | --- |
| Sex, male/female (female%) | 487/764 (61.1) | 41/140 (77.3) | <.0001^†^ |
| Age, years, mean (SD) | 58.4 (16.8) | 61.9 (15.7) | 0.0295 |
| Onset age, years, mean (SD) | 46.7 (18.6) | 49.9 (17.7) | 0.0857 |
| Disease duration, years, mean (SD) | 12.8 (9.40) | 13.5 (10.2) | 0.5007 |
| Time to beginning immunotherapy, years, mean (SD) | 1.78 (4.17) | 2.14 (3.95) | 0.2897 |
| Bulbar symptoms, n (%) * | 748 (59.8) | 117 (64.6) | 0.2234 |
| MG crisis onset, n (%) * | 119 (9.51) | 16 (8.84) | 0.8918 |
| EOMG/LOMG/TAMG, (%) ** | 37.1/32.9/30.0 | 40.0/35.8/24.2 | 0.3151 |
| AChR-Ab positivity, n (%) * | 1101 (88.0) | 155 (85.6) | 0.3959 |
| MuSK-Ab positivity, n (%)* | 14 (1.12) | 6 (3.31) | 0.0112 |
| Thymoma, n (%) * | 375 (30.0) | 44 (24.3) | 0.1371 |
| Thymectomy, n (%) * | 736 (58.8) | 104 (57.5) | 0.7470 |
| Current MG-ADL, mean (SD) | 1.05 (1.20) | 2.78 (1.75) | <.0001^†^ |
| Current QMG, mean (SD) | 3.70 (2.49) | 7.65 (2.91) | <.0001^†^ |
| Current cMG-QOL15, mean (SD) | 0.13 (0.16) | 0.28 (0.22) | <.0001^†^ |
| Worst MGFA class, (II/III/IV/V), % ** | 63.7/20.1/5.91/9.51 | 53.6/32.0/5.56/8.84 | 0.0155 |
| Maximum dose of PSL, mg, mean (SD) | 25.9 (19.8) | 22.5 (18.7) | 0.0460 |
| Current dose of PSL, mg, mean (SD) | 3.37 (3.77) | 3.66 (3.70) | 0.2545 |
| CNI use, n (%) * | 711 (56.8) | 117 (64.6) | 0.0531 |
| IVIg use, n (%) * | 205 (16.4) | 34 (18.8) | 0.4550 |
| Plasmapheresis use, n (%) * | 409 (32.7) | 58 (32.0) | 0.9324 |
| Worst QMG, mean (SD) | 13.5 (6.47) | 15.7 (7.03) | 0.0004^†^ |
| Worst MGC, mean (SD) | 15.7 (10.3) | 16.8 (10.0) | 0.1650 |

*MG*, myasthenia gravis; *MM-or-better*, minimal manifestations-or-better status; *MGC*, myasthenia gravis composite scale; *AChR-Ab*, anti-acetylcholine receptor antibody; *CNI*, calcineurin inhibitor; *EOMG*, early-onset myasthenia gravis; *IVIg*, intravenous immunoglobulin at 0.4 g/kg/day for 5 days; *LOMG*, late-onset myasthenia gravis; *MG-ADL*, myasthenia gravis activities of daily living scale; *MGFA*, Myasthenia Gravis Foundation of America; *MuSK-Ab*, anti-muscle-specific kinase antibody; *PSL*, prednisolone; *QMG*, quantitative myasthenia gravis score; *SD*, standard deviation; *cMG-QOL15*, corrected 15-item myasthenia gravis quality of life scale; *TAMG*, thymoma-associated myasthenia gravis. *** Fisher’s exact test, **** chi-square test, ^†^p < .002 for Bonferroni correction.
